# 细菌外膜囊泡亚群的分离与蛋白质组学分析

**DOI:** 10.3724/SP.J.1123.2024.10028

**Published:** 2025-05-08

**Authors:** Poju YU, Xun ZOU, Yan WU, Suntao LI, Hua XIAO

**Affiliations:** 微生物代谢全国重点实验室, 上海交通大学生命科学技术学院, 上海 200240; State Key Laboratory of Microbial Metabolism, School of Life Sciences and Biotechnology, Shanghai Jiao Tong University, Shanghai 200240, China

**Keywords:** 外膜囊泡亚群, 大肠杆菌, 铜绿假单胞菌, 密度梯度离心, 蛋白质组学, outer membrane vesicle subpopulation, *Escherichia coli*, * Pseudomonas aeruginosa*, density gradient centrifugation, proteomics

## Abstract

外膜囊泡(OMVs)是革兰氏阴性细菌分泌的20~400 nm的具膜囊泡状小体,在毒性物质传递和免疫逃逸等过程中发挥重要功能。尽管许多研究已经揭示了OMVs的关键作用,但是OMVs自身存在的异质性限制了我们对其蛋白质组成和功能的深入研究。因此,对OMVs异质性亚群的组成及其生物学功能开展研究具有重要意义。本研究采用超速离心结合密度梯度离心,对大肠杆菌DH5α和铜绿假单胞菌PAO1分泌的OMVs进行了系统的分离和表征,并结合定量蛋白质组学技术对其进行了全面分析。首先,我们对超速离心得到的两种菌株OMVs粗提物进行了碘克沙醇密度梯度离心,得到了F1~F6 6个组分。接着,通过纳米颗粒追踪分析,确认了DH5α-OMVs和PAO1-OMVs粒径分布,DH5α-OMVs各亚群的平均粒径为131.0~161.0 nm, PAO1-OMVs各亚群的平均粒径为140.0~169.0 nm;通过透射电子显微镜观察到囊泡呈经典的茶托结构;蛋白质的银染和免疫印迹结果共同验证了密度梯度组分中OMVs亚群的分布,同时确定了DH5α-OMVs和PAO1-OMVs亚群的有效组分分别为F1a~F4a和F1b~F5b。然后,我们从DH5α-OMVs和PAO1-OMVs的各亚群中分别鉴定到2388种和905种蛋白质。通过k-means聚类和基因本体(GO)富集分析,我们揭示了不同密度亚群在能量代谢、物质运输、核糖体合成等生物学功能上的异质性。最后,通过对大肠杆菌DH5α-OMVs和铜绿假单胞菌PAO1-OMVs亚群的比较分析,我们发现两种菌株虽然在OMVs的基本功能上具有共性,但在各自的亚群中也展现出了不同的功能特征。DH5α-OMVs的F1a亚群富集了与氨基酸代谢和蛋白质合成相关的功能,而PAO1的F2b亚群则表现出显著的生物大分子合成功能。本研究揭示了细菌OMVs亚群具有不同的生物学功能,进而为理解细菌的致病机制和与宿主的相互作用提供新的理论基础,有利于拓展其生物学应用。

## Abstract (en)

Outer membrane vesicles (OMVs) are 20-400 nm in size, membrane-bound, and secreted by gram-negative bacteria. OMVs play important roles in processes such as toxin delivery and immune evasion. Although many studies have revealed the critical roles played by OMVs, their heterogeneity has limited our ability to attain a comprehensive understanding of their protein compositions and functions. Therefore, studying the compositions of heterogeneous OMVs subpopulations and their biological functions is important. Herein, we used ultracentrifugation combined with density-gradient centrifugation and quantitative proteomics to systematically separate, characterize, and comprehensively analyze OMVs secreted by *Escherichia coli* DH5α and *Pseudomonas aeruginosa* PAO1. First, crude OMVs extracts from both strains were obtained by ultracentrifugation and subjected to iodixanol density-gradient centrifugation to afford six fractions each. DH5α-OMVs and PAO1-OMVs particle-size distributions were then determined via nanoparticle tracking analysis, with average particle sizes of 131.0-161.0 and 140.0-169.0 nm determined for the two subpopulation, respectively. Vesicles were observed to have classical chattel structures by transmission electron microscopy. OMVs subpopulation distributions in the density-gradated fractions were determined by silver staining and protein immunoblotting, which also identified F1a-F4a and F1b-F5b as the effective DH5α-OMVs and PAO1-OMVs subpopulation fractions, respectively. We then identified 2388 and 905 proteins from the DH5α-OMVs and PAO1-OMVs subpopulation, respectively, and used k-means clustering and gene ontology (GO) enrichment analyses to reveal the heterogeneities of the various density subpopulations in terms of biological functions, such as energy metabolism, material transport and ribosome synthesis. Comparative analysis of the *E. coli* DH5α-OMVs and *P. aeruginosa* PAO1-OMVs subpopulations finally revealed that they exhibit different functional characteristics, despite sharing commonalities in their basic OMVs functions. The F1a DH5α-OMVs subpopulation was found to be enriched for functions related to amino-acid metabolism and protein synthesis, while the F2b PAO1-OMVs subpopulation exhibited significant biomolecule synthesis functions. This study revealed that bacterial OMVs subpopulations have distinct biological functions, which in turn provides a new theoretical basis for understanding the pathogenic mechanisms of bacteria and their interactions with the host, thereby expanding their biological applications.

20世纪60年代,科学家首次通过电镜发现革兰氏阴性菌的细菌细胞外囊泡(BEVs),并将这类革兰氏阴性菌产生的BEVs称为外膜囊泡(OMVs)^[1]^。OMVs是直径为20~400 nm的具膜囊泡,携带各种重要生物分子,包括脂多糖、蛋白质、核酸、脂质和毒力因子等^[2]^。OMVs在细菌-细菌和细菌-宿主的相互作用中发挥重要作用,已被广泛应用于疫苗开发、药物递送、标志物诊断和免疫调节等领域^[3⇓-5]^。因此,对OMVs开展深入研究有助于全面理解细菌的致病机制,揭示其与宿主的相互作用。

作为细胞外囊泡家族的成员,OMVs同样存在显著的尺寸异质性和功能异质性,严重限制了对其组成和生物学功能的探究,因此亟需对OMVs的异质性亚群开展分离分析研究^[6,7]^。目前,分离OMVs的方法包括超速离心法(UC)、聚合物沉淀法、尺寸排阻色谱法和磷脂酰丝氨酸分子印迹聚合法等,其中UC是分离细胞外囊泡的“金标准”^[8,9]^。UC已被广泛应用于分离细胞器、蛋白质、核酸和病毒等颗粒^[10,11]^。虽然UC能够快速分离样品并获得OMVs,但OMVs的异质性亚群很难通过超速离心加以区分。密度梯度离心法(DGC)利用不同浓度的碘克沙醇缓冲液作为密度梯度介质,可以对不同密度的生物颗粒进行分离,再通过超速离心可以获得生物颗粒亚群。通过构建从离心管顶部到底部密度逐渐增加的碘克沙醇密度梯度,OMVs在离心过程中沉降到相应的等密度区带,从而去除大部分污染物^[12,13]^,获得异质性OMVs亚群。DGC大大提高了OMVs的纯度,在临床研究和诊断方面具有应用前景。

在微生物研究方面,大肠杆菌(*Escherichia coli*)、铜绿假单胞菌(*Pseudomonas aeruginosa*)和酿酒酵母(*Saccharomyces cerevisiae*)是具有代表性的模式微生物。这些微生物易于培养,并且遗传背景明确,因而广泛用于研究细菌生理活动和宿主-病原体相互作用,也成为研究OMVs形成和功能的重要模型^[14⇓-16]^。微生物分泌的OMVs具有异质性。例如,在多形拟杆菌中,不同生长阶段的OMVs携带的蛋白质类型和浓度不同^[17]^。因此,研究OMVs异质性对于理解其在病原性和宿主反应中的作用至关重要。本研究通过密度梯度离心将同一微生物来源的OMVs按照密度分成不同亚群组分,采用纳升液相色谱-串联质谱对OMVs亚群蛋白质组进行分析鉴定,并结合生物信息学方法探究其功能和参与的信号通路。通过密度梯度离心将OMVs分成亚群并开展研究,有助于理解不同亚群在细胞间通信、免疫调节、抗菌等方面的作用机制,为OMVs功能研究提供新线索。

## 1 实验部分

### 1.1 仪器、试剂与材料

生物型透射电子显微镜(Tecnai G2)、超净工作台、纳升液相色谱(Vanquish)和轨道离子阱质谱仪(Obitrap Exploris 480)购于赛默飞公司(美国)。超声破碎仪购于上海比朗仪器有限公司(中国), 5424离心机和离心浓缩仪购于艾本德公司(美国),涡旋振荡器购于思博明公司(美国),超速离心机(Optimal XPN-100)购于贝克曼公司(美国),全自动化化学发光和荧光成像分析系统购于上海天能科技有限公司(中国),电泳仪购于伯乐生命医学产品公司(美国),恒温培养箱购于上海慧泰仪器制造有限公司(中国),纳米颗粒追踪分析仪(NTA,ZetaView)购于Particle Metrix公司(德国)。

蛋白质酶抑制剂购于罗氏公司,5×蛋白上样缓冲液、酵母提取物、氯化钠、胰蛋白胨和蛋白质浓度测定(BCA)试剂盒购于赛默飞公司(美国)。丙三醇、盐酸(36%~38%)、氢氧化钠(≥96%)购于国药集团化学试剂有限公司(中国)。乙二胺四乙酸(EDTA)购于上海阿拉丁工业公司(中国);三羟甲基氨基甲烷(Tris)购于北京伊诺凯公司(中国)。OptiPrep密度梯度介质、尿素和ZipTip C18层析柱、过滤辅助样品制备(FASP)超滤管(10 kDa)、乙腈(色谱级)、甲酸(色谱级)购于默克公司(美国)。聚碳酸酯离心瓶26.3 mL、超净开口管13.2 mL、Type 70Ti转子和SW 41Ti转子购于贝克曼公司(美国)。10×PBS缓冲液、蔗糖、快速银染试剂盒购于上海生工生物工程公司(中国)。33 mm/0.22 μm聚偏氟乙烯(PVDF)灭菌针头过滤器购于密理博公司(美国)。脱脂奶粉、HEPES-Tris 10%预制胶、ECL化学发光超敏显色试剂盒和过氧化物酶标记的兔抗羊IgG(H+L)二抗购于上海翌圣生物科技公司(中国)。外膜蛋白A(ompA)一抗购于Antibody Research公司(美国)。

### 1.2 培养基配制

LB液体培养基:蛋白胨10 g/L、酵母提取物5 g/L、氯化钠10 g/L, pH 7.1, 115 ℃灭菌30 min。

LB固体培养基:在LB液体培养基中加入1.2%(质量分数)的琼脂粉,121 ℃灭菌20 min。

### 1.3 细菌培养

本研究选取大肠杆菌DH5α菌株和铜绿假单胞菌PAO1(微生物代谢全国重点实验室)作为模式菌株。取出保存的菌种,利用接种针在LB固体培养基上画线,37 ℃过夜培养,然后挑取单菌落至5 mL LB液体培养基中(装在15 mL离心管中), 37 ℃培养6~8 h后,全部转入200 mL LB液体培养基(装在1 L锥形瓶中), 37 ℃摇床220 r/min过夜培养。

### 1.4 样品制备

将细菌悬液于4 ℃以5000 g的转速离心10 min,去除细胞和细胞碎片。用1×PBS重悬,4 ℃以5000 g的转速离心10 min,重复一次后向沉淀加入6 mol/L尿素裂解并超声得到细菌裂解物蛋白质(Bac)。上清液在4 ℃以100000 g的转速离心70 min。用1×PBS(0.22 μm)重悬并涡旋振荡10 min,得到OMVs粗提物悬液。

配制密度梯度缓冲液A(A液): 0.25 mol/L蔗糖、6 mmol/L EDTA、60 mmol/L Tris。配制密度梯度缓冲液B(B液): 0.25 mol/L蔗糖、1 mmol/L EDTA、60 mmol/L Tris。A液和B液的pH值调节至7.4,并用0.22 μm PVDF针头过滤器过滤,4 ℃保存。配制密度梯度缓冲液C(C液): 1∶5(v/v)比例混合A液与OptiPrep。将B液、C液按表1比例混合,配制2 mL 20%、24%、28%、30%、32%、35%的密度梯度缓冲液,密度从大到小依次铺入,最上层加入150 μL OMVs样品,最后用1×PBS补体积至超离管管口2~3 mm处,4 ℃以216000 g的转速离心6 h。从上至下,每2 mL取为1个组分,共取6个组分,分别记为F1~F6,用1×PBS稀释4倍后,4 ℃以170000 g的转速离心2 h,用1×PBS重悬。

**表1:** 2 mL密度梯度缓冲液的配制

Iodixanol/%^*^	Buffer B/(mL)	Buffer C/(mL)	*ρ*/(g/mL)
20.00	1.20	0.80	1.127
24.00	1.04	0.96	1.146
28.00	0.88	1.12	1.165
30.00	0.80	1.20	1.175
32.00	0.72	1.28	1.185
35.00	0.60	1.40	1.199

* Mass fraction.

### 1.5 纳米颗粒追踪分析

使用ZetaView仪器分析细菌外膜囊泡及其亚群的尺寸分布和囊泡颗粒浓度。在分析前,注入10 mL PBS清洗检测通道,并进行校准。用1×PBS按1∶100~1∶1000的体积比稀释样品,取1 mL样品检测,每个样品重复实验3次,结果以均值±标准差显示。仪器温度设置为25.0 ℃,每秒帧数设置为30,测量时间为60 s,激光波长设置为488 nm的单色激光束。

### 1.6 透射电子显微镜检测

首先,亲水处理铜网5 min。其次,将OMVs粗提物和富集的OMVs亚群重悬在20 μL双蒸水中,用双蒸水稀释5~8倍,滴在经过亲水处理的铜网上,静置1 min,用滤纸贴近铜网,将多余液体吸除。用双蒸水清洗后,再用4 μL 2%磷钨酸(pH值6.5~7.0)负染色45 s,用滤纸将染液吸走。最后,待铜网干燥后使用透射电子显微镜进行形态检测。

### 1.7 蛋白质免疫印迹

向OMVs及其亚群中加入6 mol/L尿素,冰水浴超声1 min(超声5 s,停3 s),并以体积比1∶100加入蛋白酶抑制剂,在冰上裂解1 h得到蛋白质组分。经过BCA法测定蛋白质浓度后,取20 μg蛋白质样品,加入5×蛋白质上样缓冲液混匀,99 ℃加热10 min。用HEPES-Tris 10%预制胶150 V电压下电泳30 min,恒流300 mA电泳1 h将蛋白质转印至PVDF膜。室温封闭1 h后,4 ℃过夜孵育ompA一抗(以1∶1000的体积比含0.1%吐温20的磷酸盐缓冲液(PBST)稀释)。经PBST洗涤3次后,室温孵育过氧化物酶标记的兔抗羊IgG(H+L)二抗(以1∶10000的体积比用PBST稀释)。洗涤3次后,加入ECL化学发光超敏显色试剂,并用全自动化学发光和荧光成像分析系统拍照,曝光时间30 s。

### 1.8 十二烷基硫酸钠聚丙烯酰胺凝胶银染

取2 μg OMVs及其亚群的蛋白质,经1.7节同样条件电泳后用蒸馏水冲洗,然后按照快速银染试剂盒的操作说明进行银染。

### 1.9 纳升液相色谱-串联质谱分析

取20 μg经过还原烷基化的蛋白质样品,按照常规FASP方法进行酶解,蛋白质样品与胰酶的质量比为50∶1。37 ℃酶解16 h后,通过ZipTip C18柱除盐。取500 ng除盐后的肽段上样进行纳升液相色谱质谱-串联质谱分析,每个样品进行3次技术重复。

液相色谱的相关参数如下:自填C18-AQ填料分析柱(20 cm×75 μm, 3 μm);流动相A为0.1%甲酸水溶液,流动相B为含0.1%甲酸的80%乙腈水溶液;梯度洗脱(0~1 min, 4%B~5%B; 1~29 min, 5%B~16%B; 29~52 min, 16%B~32%B; 52~53 min, 32%B~99%B; 53~60 min, 99%B)。流速为300 nL/min,色谱柱温度为室温,进样体积为2 μL。

串联质谱的相关参数如下:电喷雾毛细管电压1900 V;气体流速4.0 L/min;离子传输管温度为280 ℃。质谱仪使用高能碰撞解离(HCD)的碎裂方法,采用数据依赖扫描(DDA)程序模式,全扫描MS^1^分辨率为60000; *m/z*范围为350~1750;归一化自动增益控制(AGC)目标值为300%;最大注入时间为25 ms; MS^2^分辨率为15000;归一化AGC目标值为75%;最大注入时间为22 ms;隔离窗口为*m/z* 1.6;动态排除时间为45 s。当使用高场非对称波形离子迁移谱(FAIMS)时,补偿电压设定为-45 V和-65 V;射频透镜(RF lens)设定为50%。

### 1.10 生物信息学分析

使用Proteome Discoverer(版本3.0)对采集的数据进行分析,检索*Escherichia coli*蛋白质数据库(Uniprot,版本2024_04_01, 9028396条序列)和*Pseudomonas aeruginosa*蛋白质数据库(Uniprot,版本2024_04_22, 544901条序列)。数据库检索设置允许母离子质量误差值为1.0×10^-5^(10 ppm),漏切位点最大值为2,蛋白质和肽段水平的鉴定假阳性(FDR)小于1%。蛋白质鉴定标准为检测到至少1个特征肽段,蛋白质及二级谱图数量(PSM)的假阳性率为小于1%。以四分位法对蛋白质表达量值(Abundance)进行标准化和*z*-score归一化。

对蛋白质表达量用k-means聚类算法对亚群进行聚类,通过基因本体论(GO)数据库注释每个聚类中富集到基因行使的分子功能(MF)和参与的生物学过程(BP)(*p*<0.05),通过R包clusterGVis将结果可视化。借助微生信网站(https://www.bioinformatics.com.cn)绘制Venn图。

利用STRING网站(https://cn.string-db.org/)进行蛋白质互作网络分析,输入候选蛋白质的ID后,选择铜绿假单胞菌作为物种,将最低要求的交互置信度(minimum required interaction score)设置为0.400。

## 2 结果与讨论

### 2.1 两个模式菌种OMVs亚群的分离纯化与表征

#### 2.1.1 大肠杆菌DH5α-OMVs的分离与表征

我们通过UC提取了大肠杆菌DH5α菌株的OMVs粗提物(DH5α-OMVs),通过NTA和TEM检测了DH5α-OMVs的粒径分布和囊泡形态。NTA结果如图1a所示,DH5α-OMVs的平均粒径为(141.0±5.0) nm,平均含量为(6.0±0.8)×10^11^ particles/mL。通过TEM观察DH5α-OMVs的形态特征,TEM结果如图1b所示,图中箭头指出的中间暗周围亮的圆形或椭圆形结构呈杯托状,是OMVs经典的形态特征,说明粗提物中含有囊泡。因此,我们继续利用选定的密度梯度参数通过DGC将DH5α-OMVs分离成6个密度不同的组分,分别为F1a~F6a。

**图1:**
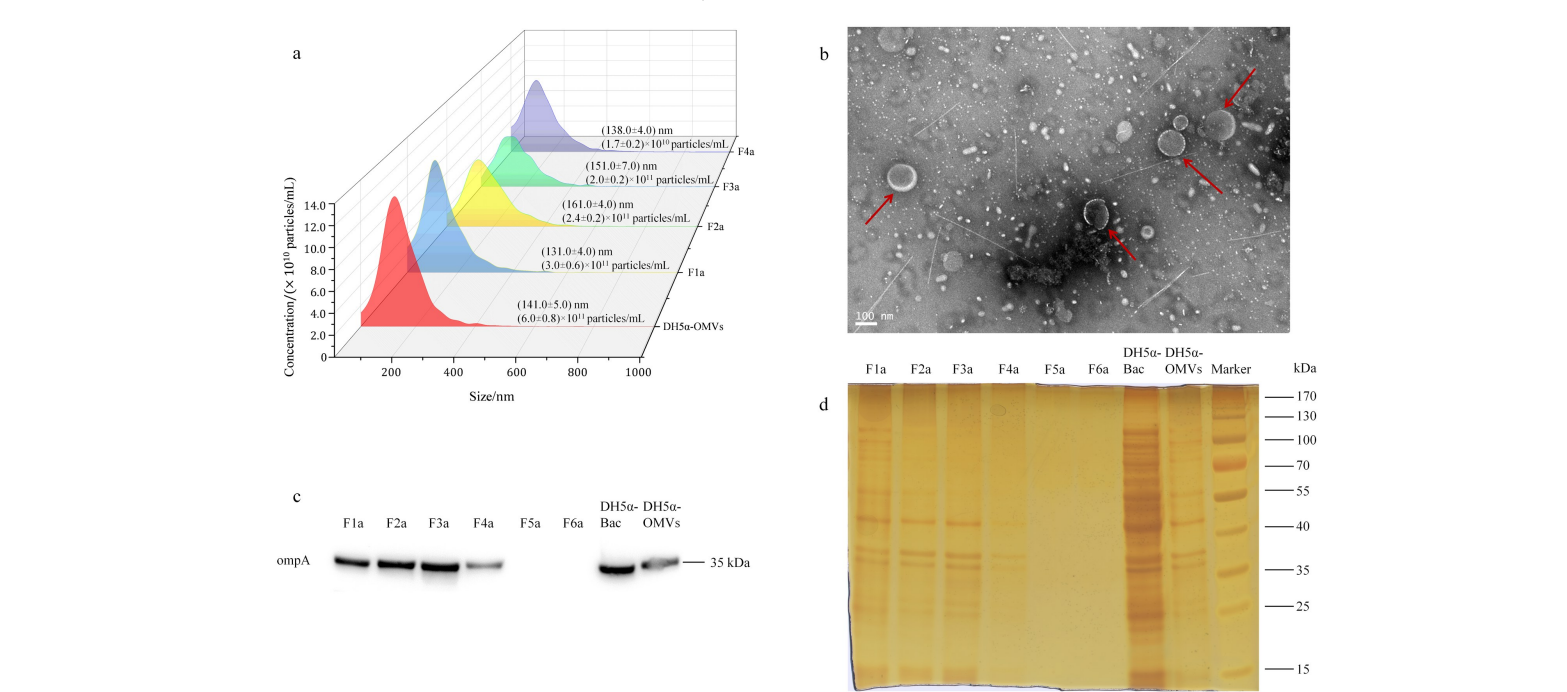
DH5α-OMVs和F1a~F6a亚群的表征

为了确定DH5α-OMVs存在于哪些组分中,我们用WB检测了革兰氏阴性菌特征蛋白标志物ompA。如图1c所示,在F1a~F4a、大肠杆菌细胞裂解液DH5α-Bac和DH5α-OMVs中均检测到目标条带,但在F5a、F6a中未检测到,可能原因是F5a、F6a组分的蛋白质浓度太低。为了验证这一点,我们对F1a~F6a做了蛋白质银染检测,结果如图1d所示,F1a组分蛋白质条带与OMVs相似度较高,组分F2a与F3a蛋白质条带几乎一致,而组分F4a蛋白质条带较浅,F5a、F6a几乎没有蛋白质条带。这一结果与WB表征结果相吻合,因此我们认为组分F1a~F4a为分离得到的大肠杆菌OMVs亚群。接着,我们通过NTA检测了不同密度的DH5α-OMVs亚群的粒径分布,结果如图1a所示,F1a~F4a的平均粒径分别为(131.0±4.0)、(161.0±4.0)、(151.0±7.0)和(138.0±4.0) nm,说明不同密度亚群的平均粒径存在差别。同时,随着密度逐渐增大,F1a~F4a中OMVs的颗粒浓度逐渐降低。

#### 2.1.2 铜绿假单胞菌PAO1-OMVs的分离与表征

我们通过UC提取了铜绿假单胞菌PAO1菌株的OMVs粗提物(PAO1-OMVs)。通过TEM和NTA检测PAO1-OMVs的形态特征和粒径分布。NTA结果如图2a所示,PAO1-OMVs的平均粒径为(158.0±15.0) nm,平均含量为(8.9±3.5)×10^12^ particles/mL。TEM结果如图2b所示,PAO1-OMVs呈杯托结构,也符合OMVs的典型形态特征。接着,我们利用相同的密度梯度将PAO1-OMVs分离成6个密度不同的组分,分别为F1b~F6b。

**图2:**
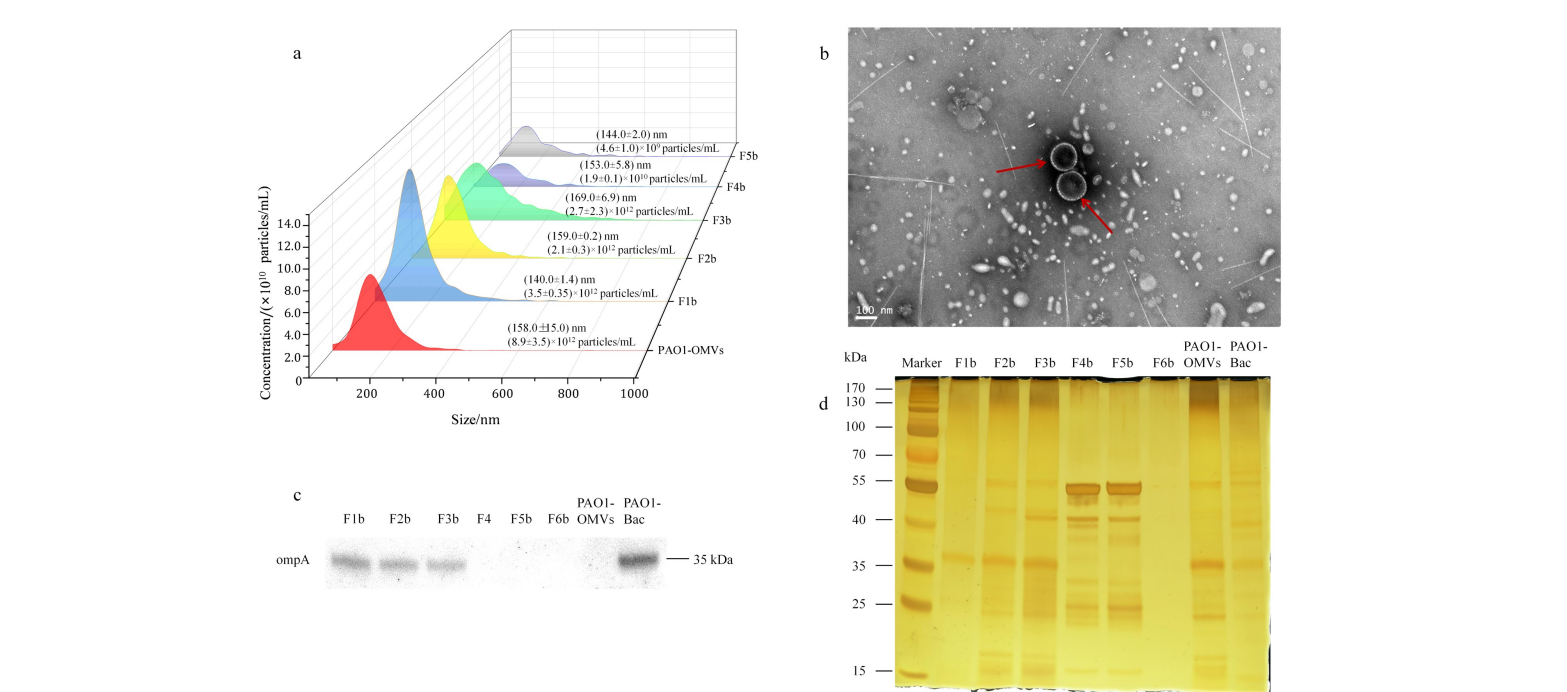
PAO1-OMVs以及F1b~F6b亚群的表征

为了确定PAO1-OMVs存在于哪些组分中,我们通过WB检测了ompA,结果如图2c所示,在F1b~F3b和铜绿假单胞菌系细胞裂解物PAO1-Bac中均检测到目标条带,而在F4b~F6b和PAO1-OMVs中未检测到。对比图2d所示的蛋白质银染结果,与大肠杆菌不同,铜绿假单胞菌的银染结果显示不同组分之间的蛋白质条带差异较大,验证了不同OMVs组分在蛋白质表达上具有异质性。其中,组分F2b、F3b与OMVs条带相近;而F4b、F5b条带相近且与其他条带有较大区别,在55 kDa有较为明显的蛋白质条带,与WB结果一致。

因此,我们认为F1b~F5b为分离到的铜绿假单胞菌OMVs亚群。接着,我们通过NTA检测了不同密度PAO1-OMVs亚群的粒径分布,F1b~F5b亚群NTA结果如图2a所示,F1b~F5b的平均粒径分别为(140.0±1.4)、(159.0±0.2)、(169.0±6.9)、(153.0±5.8)和(144.0±2.0) nm,符合OMVs的粒径特征。在F4b和F5b组分中,OMVs的颗粒浓度较其他组分明显偏少。

### 2.2 两类模式菌种不同密度OMVs亚群的蛋白质组分析

#### 2.2.1 DH5α-OMVs及其亚群的蛋白质组分析

图3a为DH5α-OMVs全蛋白质组的韦恩图,在DH5α-OMVs粗提物和4个DH5α-OMVs亚群中分别鉴定到2116和2364种蛋白质,其中共有的蛋白质达到2092种,占所有检测到蛋白质的87.6%。通过密度梯度离心的分离方法,我们从4个DH5α-OMVs亚群中鉴定到了272种独有蛋白质。图3b为F1a~F4a亚群的全蛋白质组韦恩图,分别鉴定到了来源于F1a、F2a、F3a和F4a亚群的2073、2096、2093和1850种蛋白质,其中共有的蛋白质达到1689种,占总DH5α-OMVs亚群2364种蛋白质的71.4%。在F1a、F2a、F3a和F4a亚群中分别鉴定到了72、29、118和21种独有蛋白质,这表明不同密度的DH5α-OMVs亚群具有独特的蛋白质组成,可能反映了不同密度亚群DH5α-OMVs在功能上的异质性。

**图3:**
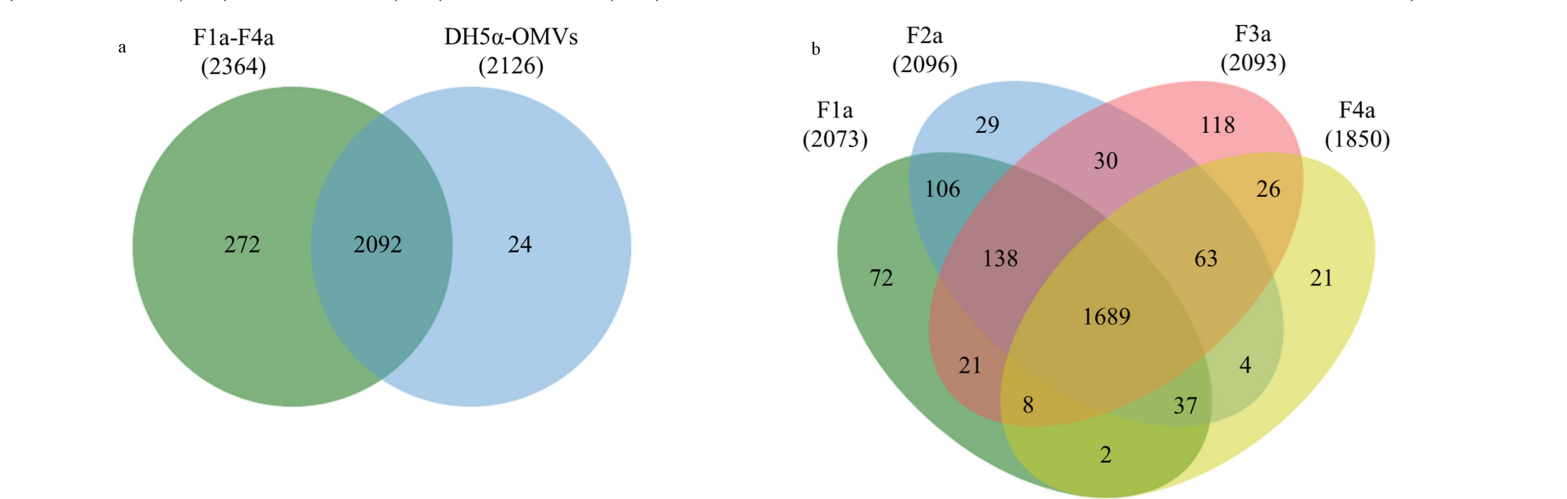
(a)DH5α-OMVs亚群的蛋白质组与DH5α-OMV总蛋白质组和(b)F1a~F4a亚群的蛋白质比较

为了进一步探索这些亚群的具体生物学功能,我们对DH5α-Bac、DH5α-OMVs及其不同密度梯度亚群根据质谱数据中蛋白质的表达量及其他相关特征,使用k-means聚类算法将蛋白质划分为不同的群集进行聚类分析,将具有相似表达模式的蛋白质分组到同一类别中。根据蛋白质聚类的结果,绘制热图来可视化不同聚类中蛋白质的表达模式。如图4所示,所有蛋白质被聚成C1~C5 5类,其中DH5α-Bac主要聚到C4类;DH5α-OMVs、F1a主要聚到C5类; F2a主要聚到C2类; F3a主要聚到C3类,且蛋白质高表达;F4a主要聚到C1类。从整体来看,DH5α-Bac中主要富集了与细菌生长和应激反应相关的功能,这些蛋白质主要集中在细胞的自我保护机制以及环境变化的适应性上。相比之下,总DH5α-OMVs的功能主要与物质的运输、细胞信号传递、代谢活动等生理过程相关。DH5α-OMVs与DH5α-Bac的共同点在于两者都具有与应激反应相关的功能,显示出DH5α-OMVs在细菌生存和适应中的重要作用。然而,与DH5α-Bac相比,DH5α-OMVs在细胞间通信和物质运输方面的功能更加突出,表明DH5α-OMVs在宿主-病原体相互作用中发挥作用。

**图4:**
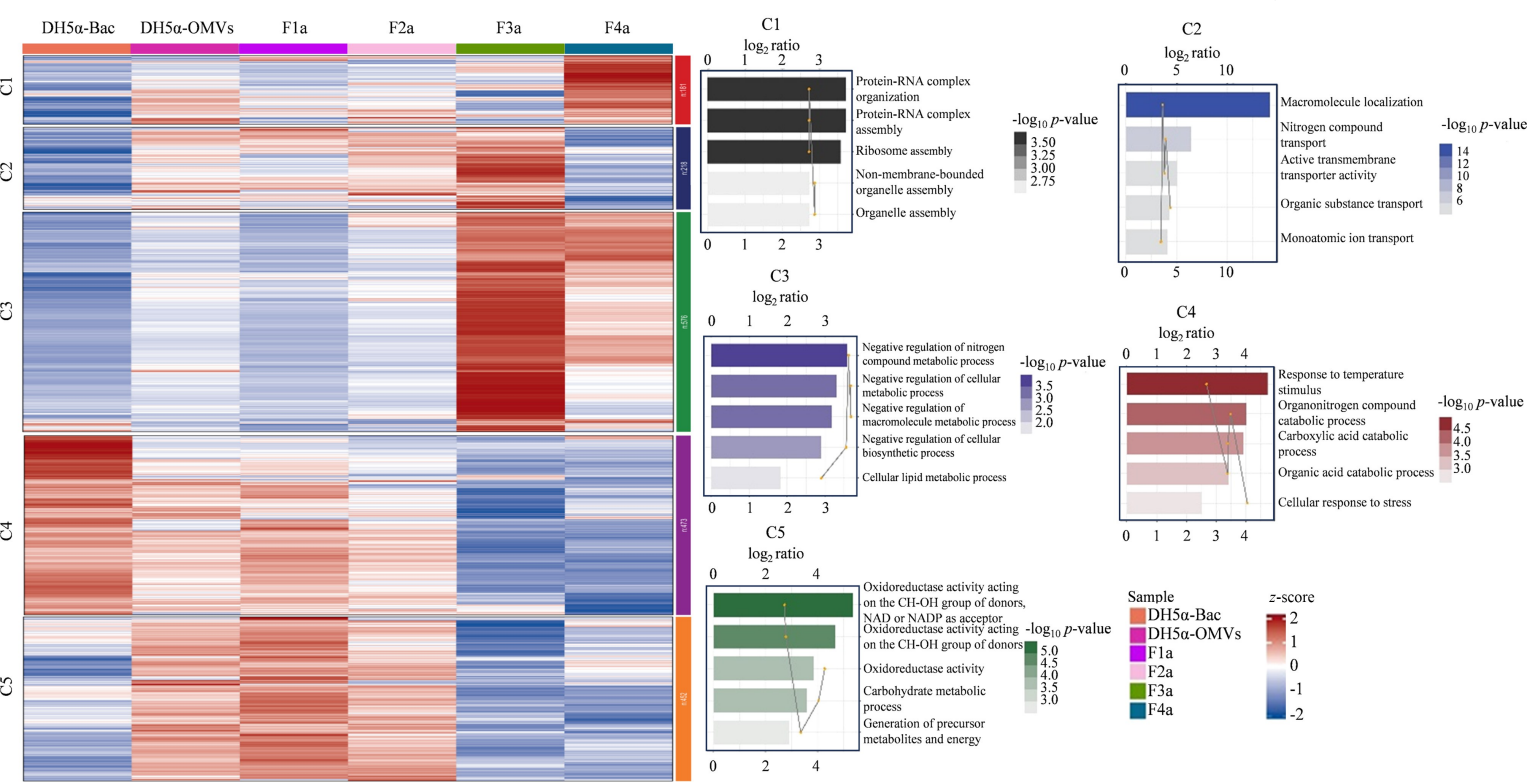
基于k-means聚类的DH5α-OMVs、DH5α-Bac和F1a~F4a亚群蛋白质功能GO注释热图(*p*≤0.05)

#### 2.2.2 PAO1-OMVs及其亚群的蛋白质组分析

通过密度梯度离心分离,我们将铜绿假单胞菌分泌的PAO1-OMVs分成了F1b~F5b亚群,图5a为PAO1-OMVs粗提物与5个PAO1-OMVs亚群全蛋白质组的韦恩图。共鉴定到905种蛋白质,其中在PAO1-OMVs粗提物和总PAO1-OMVs亚群中分别鉴定到717和897种蛋白质,其中共有的蛋白质达到709种,占所有检测到蛋白质的78.3%。我们从总PAO1-OMVs亚群中鉴定到了188种独有蛋白质,提升了PAO1-OMVs的鉴定深度。通过定量蛋白质组学分析,我们鉴定到了来源于F1b、F2b、F3b、F4b和F5b亚群的771、815、841、431和410种蛋白质,图5b为F1b~F5b亚群的全蛋白质组韦恩图,其中5个亚群共有的蛋白质340种,仅占总PAO1-OMVs亚群的37.9%,说明亚群之间蛋白质组成的差异明显。

**图5:**
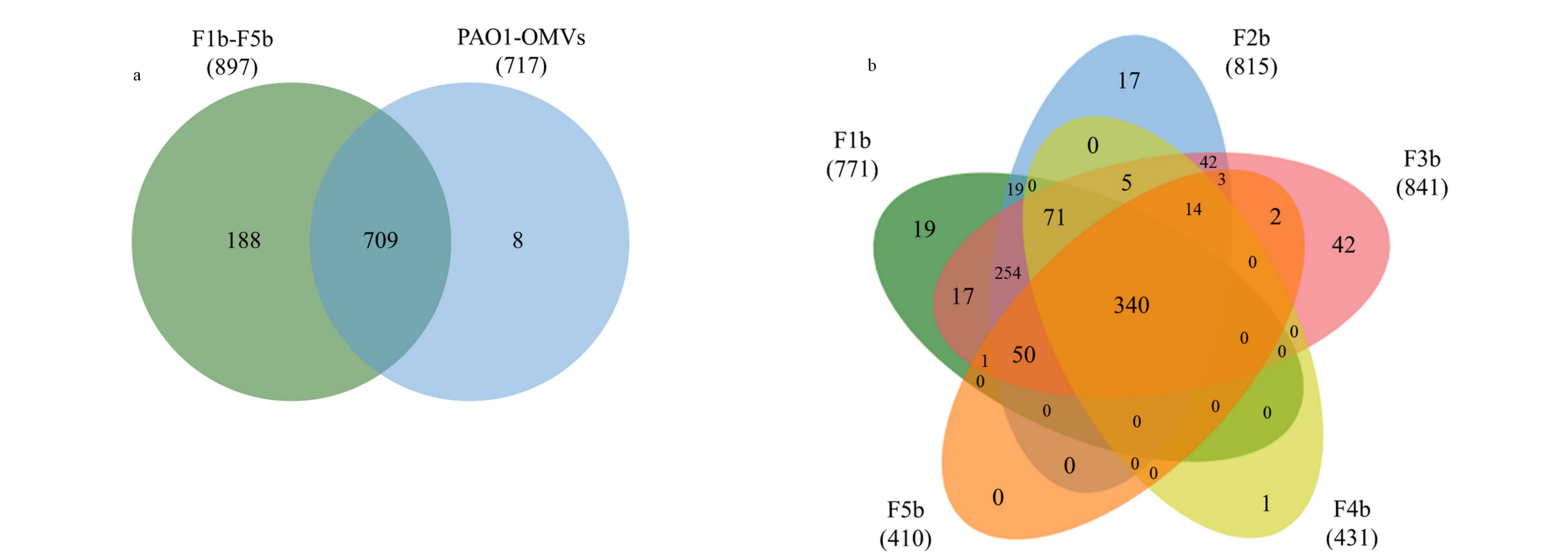
(a)PAO1-OMVs亚群的蛋白质组与PAO1-OMV总蛋白质组和(b)F1b~F5b亚群的蛋白质比较

对PAO1-Bac、PAO1-OMVs及其不同密度梯度的5个亚群进行蛋白质组学分析,生物信息学分析结果如图6所示。通过聚类分析和GO富集分析,将所有检测到的蛋白质聚成六类(C1~C6),并进行功能注释。图中展示了每个聚类中的蛋白质在不同样品中的表达情况及其主要功能。

**图6:**
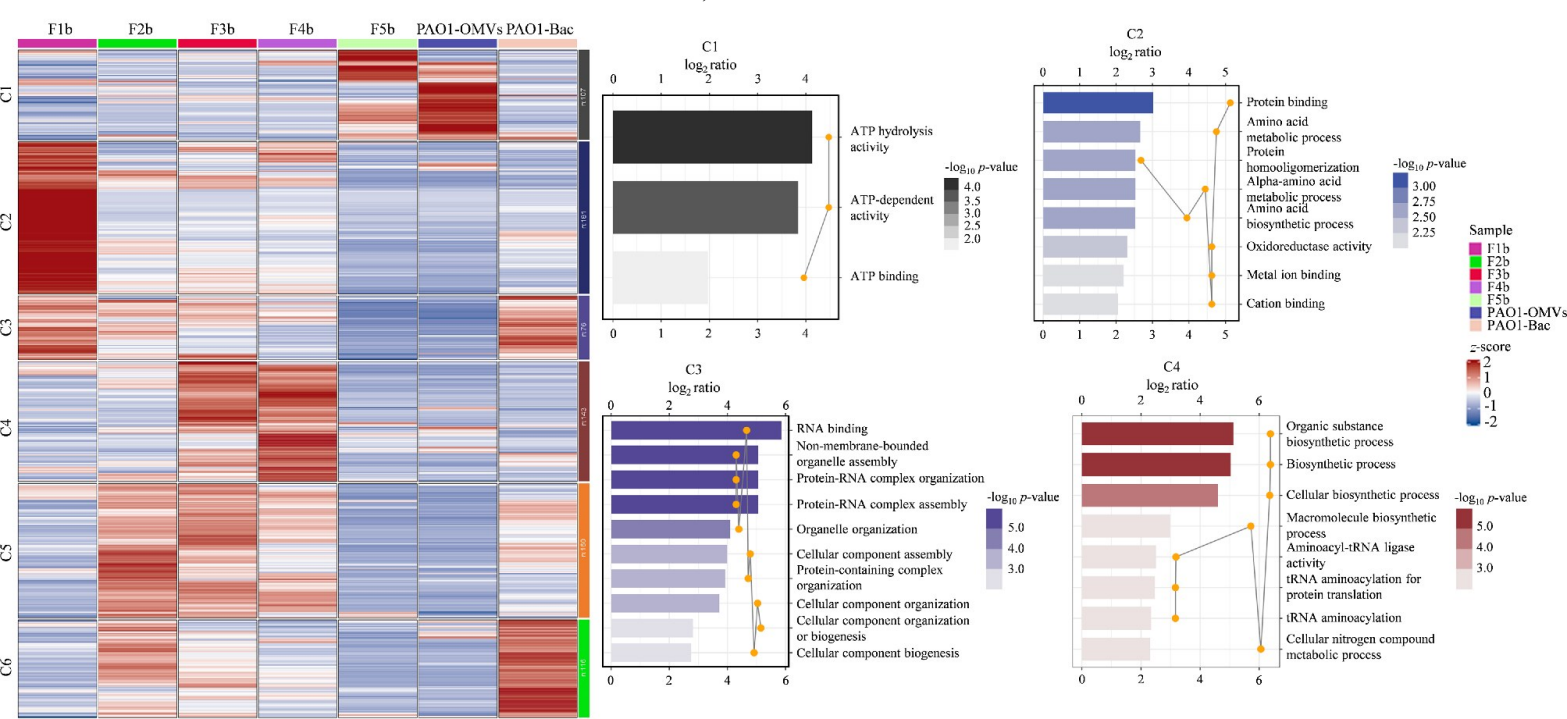
基于k-means聚类的PAO1-OMVs、PAO1-Bac和F1b~F5b亚群蛋白质功能GO注释热图(*p*≤0.05)

从整体来看,PAO1-Bac中富集了与细菌生长和基础代谢相关的功能,这些功能涉及细胞自我维持及环境适应。而总PAO1-OMVs则富集了与物质运输、细胞间通信以及宿主相互作用相关的功能。相比PAO1-Bac, PAO1-OMVs中的蛋白质与宿主细胞的交互更加密切,表明PAO1-OMVs在细菌毒力及宿主相互作用中具有重要作用。

进一步对密度梯度分离后的亚群进行分析,我们发现这些亚群在总PAO1-OMVs的基本功能上表现出一些独特的功能。C1聚类中,主要包含PAO1-OMVs和F5b亚群,C1聚类涉及三磷酸腺苷(ATP)分子的参与,是细胞内能量转化和信号传导的重要步骤。通过这些过程,细胞能够利用ATP的能量完成各种生物学功能,维持细胞的生存和正常代谢^[18]^。C2聚类主要包含F1b亚群,C2聚类的通路主要关于氨基酸和蛋白质,氨基酸的代谢过程包括氨基酸的合成、降解和转化,这些过程对于细胞的蛋白质合成和代谢调节至关重要。这些过程反映了F1b亚群在蛋白质和代谢物调控方面的重要作用,特别是在细胞适应环境变化和维持内稳态方面的功能。C4聚类主要包含F3b和F4b亚群,主要涉及细胞内复合物和结构的形成,包括核糖体等细胞器以及组织和调控过程,是维持细胞内结构和功能的重要机制。C5聚类主要包含F2b亚群,其高表达的蛋白质涉及有机物和生物大分子的合成过程,如tRNA的氨酰化过程。这些功能与细胞内生物大分子的合成及其后续功能的实现密切相关,表明F2b亚群在蛋白质翻译和细胞生物合成中具有重要作用^[19]^。通过对PAO1-OMVs不同密度亚群的详细分析,我们发现每个亚群都具有特定的功能富集。这为进一步理解PAO1-OMVs亚群在细菌致病机制、宿主相互作用及环境适应中的作用提供了重要线索。

### 2.3 两种细菌OMVs亚群蛋白质组的比较分析

通过对DH5α-OMVs和PAO1-OMVs以及它们不同密度梯度亚群的蛋白质组学分析,我们发现两种菌株在OMVs组分中都富集了与ATP水解活性、ATP依赖活动和ATP结合相关的蛋白质,表明两者在能量代谢和信号传导方面具有共性。ATP在OMVs的生物合成和动力学中发挥关键作用,可能在相似的生理环境中支持细菌的能量需求和生存适应^[20]^。

两种菌株的OMVs亚群蛋白质组也存在部分相似的和差异较大的生物学功能。例如在DH5α的F1a亚群中,蛋白质同源寡聚化过程尤为显著,这反映了该亚群在调控蛋白质互作和形成多蛋白质复合物方面的重要性。PAO1的F2b亚群主要富集到与有机物和生物大分子合成过程相关的蛋白质,例如tRNA的氨酰化过程,影响着蛋白质合成的准确性和效率。莫匹罗星是一种已经被批准用于氨酰-tRNA合成酶的选择性抑制剂,通过阻碍蛋白质合成从而实现抗菌,而在OMVs中富集到相关功能的蛋白质可能有助于对抗药性的新认识^[21]^。PAO1的F3b亚群更多地富集到与rRNA结合、核糖体组装和细胞器组织相关的通路,这些过程反映了该亚群在蛋白质合成和细胞内结构功能维持中的重要作用。在两种菌株的F4a和F4b亚群中,均富集到与核糖体合成以及rRNA结合相关的通路,显示出它们在蛋白质合成和核糖体结构维持方面的共同功能,凸显了这些功能在细菌细胞生命活动中的核心作用。密度梯度分离后的亚群展现了比总OMVs更加精细的功能分化,这也表明亚群分离有助于揭示OMVs中存在的异质性功能及其在细菌生命活动中的特定角色。

根据OMVs不同亚群的蛋白质组数据,我们比较了DH5α-OMVs和PAO1-OMVs中同源蛋白质的功能,发现了7种与应激响应相关的蛋白质,这些蛋白质在两种微生物的某些OMVs亚群中得到了显著富集(OMVs亚群/OMVs粗提物≥1.5)。如表2所示,ClpB、DnaJ、DnaK、GrpE和HtpG与热休克蛋白家族相关^[22⇓-24]^,其中,伴侣蛋白DnaK是蛋白质折叠过程中的一个关键成分,主要富集于DH5α-OMVs和PAO1-OMVs的F1a和F1b亚群,而细菌应激反应性伴侣蛋白HtpG则主要富集于DH5α-OMVs的F1a、F2a亚群和PAO1-OMVs的F5b亚群。

**表2:** DH5α-OMVs亚群和PAO1-OMVs亚群中富集到的与应激响应相关的同源蛋白质

Gene	Protein name	Enriched in DH5α-OMVs subpopulations	Enriched in PAO1-OMVs subpopulations
*clpB*	chaperone protein ClpB	F1a	F3b, F4b
*dnaJ*	chaperone protein DnaJ	F3a	F1b, F5b
*dnaK*	chaperone protein DnaK	F1a	F1b
*grpE*	protein GrpE	F1a	F2b, F3b
*hchA*	protein/nucleic acid	F1a	F2b, F3b
	deglycase HchA		
*hfq*	RNA-binding protein Hfq	F3a	F1b, F2b
*htpG*	chaperone protein HtpG	F1a, F2a	F5b

对这些蛋白质进行蛋白质互作网络分析,结果如图7所示,除了HchA外,其余蛋白质之间存在相互作用。在细菌OMVs亚群中鉴定到与应激响应相关蛋白质,意味着细菌可能会通过OMVs携带的这些蛋白质进行细胞间交流,并激活受体细胞以应对微环境。

**图7:**
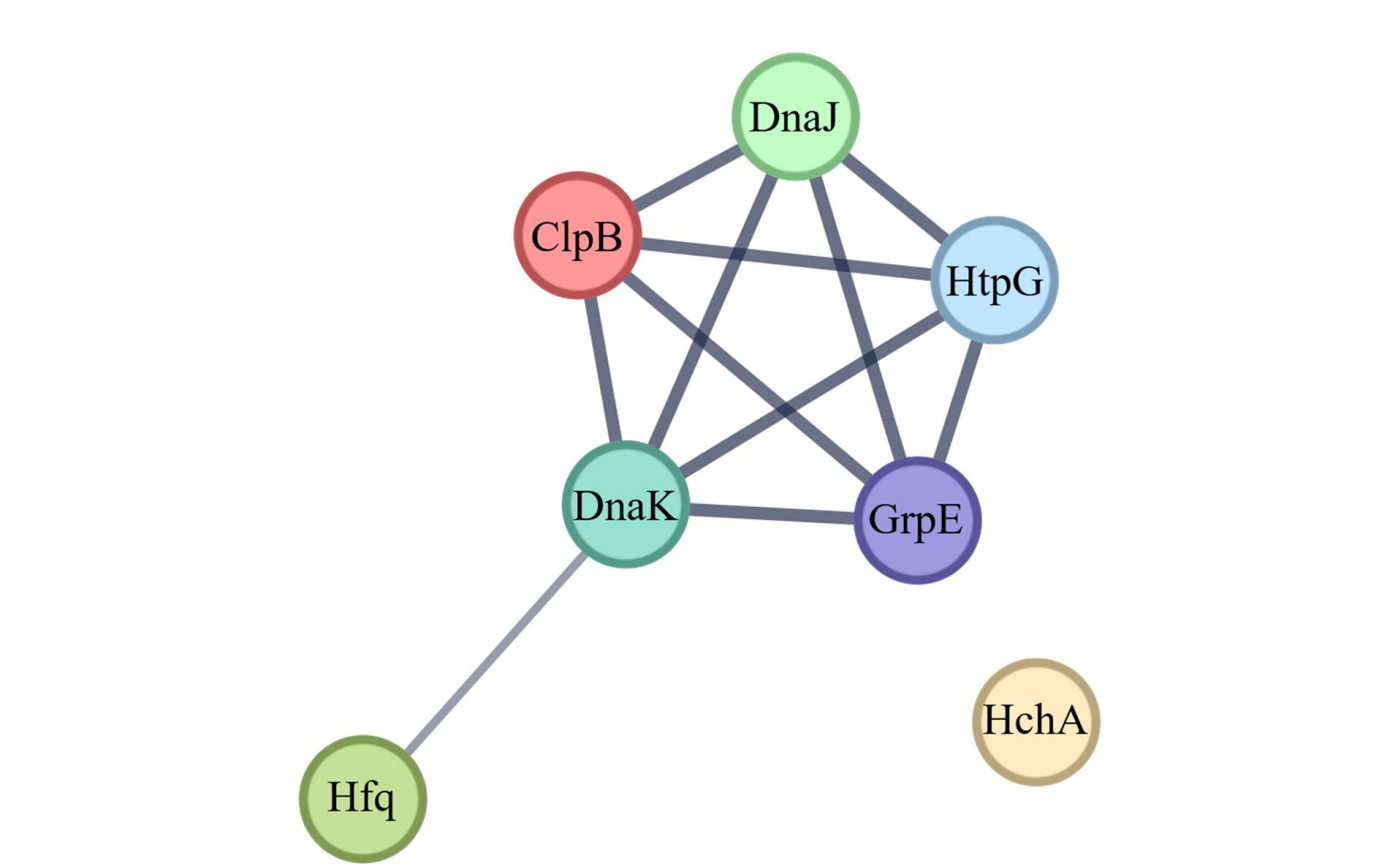
DH5α-OMVs和PAO1-OMVs中与应激响应相关的同源蛋白质互作网络图

## 3 结论

本研究通过超速离心结合密度梯度离心方法对大肠杆菌DH5α和铜绿假单胞菌PAO1分泌的OMVs进行了分离、制备和表征,并对获得的OMVs亚群进行了蛋白质组学分析,揭示了两种细菌OMVs亚群的功能特征。研究结果表明,不同密度梯度的OMVs亚群在能量代谢、物质运输、核糖体合成等方面表现出显著的功能异质性。本研究为探索不同密度OMVs亚群的生物学功能提供了重要依据,加深了对细菌OMVs的理解,也为基于OMVs的细菌功能研究和实际应用提供了新的线索。
